# Effectiveness of an Ultrasound Training Module for Internal Medicine Residents

**DOI:** 10.1186/1472-6920-11-75

**Published:** 2011-09-28

**Authors:** Mira T Keddis, Michael W Cullen, Darcy A Reed, Andrew J Halvorsen, Furman S McDonald, Paul Y Takahashi, Anjali Bhagra

**Affiliations:** 1 Division of Nephrology, Mayo Clinic, 200 First Street SW, Rochester, MN, 55905, USA; 2 Division of Cardiovascular Diseases, Mayo Clinic, 200 First Street SW, Rochester, MN, 55905, USA; 3 Division of General Internal Medicine, 200 First Street SW, Rochester, MN, 55905, USA; 4 Division of Community Internal Medicine, 200 First Street SW, Rochester, MN, 55905, USA; 5 Department: Internal Medicine Residency Office of Educational Innovations, Mayo Clinic, 200 First Street SW, Rochester, MN, 55905, USA

## Abstract

**Background:**

Few internal medicine residency programs provide formal ultrasound training. This study sought to assess the feasibility of simulation based ultrasound training among first year internal medicine residents and measure their comfort at effectively using ultrasound to perform invasive procedures before and after this innovative model of ultrasound training.

**Methods:**

A simulation based ultrasound training module was implemented during intern orientation that incorporated didactic and practical experiences in a simulation and cadaver laboratory. Participants completed anonymous pre and post surveys in which they reported their level of confidence in the use of ultrasound technology and their comfort in identifying anatomic structures including: lung, pleural effusion, bowel, peritoneal cavity, ascites, thyroid, and internal jugular vein. Survey items were structured on a 5-point Likert scales (1 = extremely unconfident, 5 = extremely confident).

**Results:**

Seventy-five out of seventy-six interns completed the pre-intervention survey and 55 completed the post-survey. The mean confidence score (SD) increased to 4.00 (0.47) (p < 0.0001). The mean (SD) comfort ranged from 3.61 (0.84) for peritoneal cavity to 4.48 (0.62) for internal jugular vein. Confidence in identifying all anatomic structures showed an increase over the pre-intervention means (p < 0.002).

**Conclusion:**

A simulation based ultrasound learning module can improve the self-reported confidence with which residents identify structures important in performing invasive ultrasound guided procedures. Incorporating an ultrasound module into residents' education may address perceived need for ultrasound training, improve procedural skills, and enhance patient safety.

## Background

Hospitalists and internal medicine residents perform bedside invasive procedures such as thoracentesis, paracentesis, and central venous catheter (CVC) placement in many teaching hospitals in the United States. Data suggests that the use of ultrasound guidance during these procedures can decrease complications, improve procedure related outcomes, and enhance patient safety [1-3]. However, many internal medicine residents lack formal training in ultrasound use.

The use of ultrasound to perform invasive procedures has been shown to be effective. Sonographic imaging enables delineation of structural abnormalities and tissue planes better than superficial anatomic landmarks, allowing invasive procedures to be performed with fewer complications [4]. Real time ultrasound guidance was shown to be the most important factor in lowering the rate of iatrogenic pneumothorax [1]. In one study, ultrasound guided thoracentesis decreased the rate of iatrogenic pneumothorax by 54% [5]. Likewise, ultrasound guided CVC placement decreases the number of arterial punctures and attempts [6] and is generally recommended for central vein cannulation [2]. In a cross-sectional study examining residents' comfort with performing bedside procedures, residents reported that the use of ultrasound was one of the most important factors to avoid procedural complications [7]. Thus, the use of ultrasound during invasive procedures has become the standard of care at many institutions in order to promote patient safety.

In addition to the multiple applications and effectiveness of ultrasound, evidence suggests that training residents and hospitalists in the use of ultrasound is feasible and successful. After a brief educational intervention, internal medicine residents were able to correctly perform and interpret the results of ultrasound screening of abdominal aortic aneurysms [8]. Likewise, emergency medicine residents demonstrated appropriate use of ultrasonography to detect acute deep venous thrombosis following a 90 minute didactic and hands-on session on ultrasound technique taught by senior vascular technicians [9]. Similarly, hospitalists were educated on the use of hand-held ultrasound machines to aid in cardiac examination and were found to have increased accuracy in detecting left ventricular failure, cardiomegaly, and pericardial effusion when compared with expert cardiologists [10].

Literature on ultrasound training workshops suggests different models of teaching ultrasound-guided CVC placement and thoracentesis. Anesthesia physicians' comfort and use of ultrasound for central vein cannulation increased after a training workshop using varying homemade models of rubber tubes mimicking vessels [11]. Pulmonologist demonstrated competence in ultrasound-guided thoracentesis after completing a half-day workshop utilizing cadaveric and inanimate models under supervision, while doing a minimum of 10 procedures on real patients [12]. Data regarding models for teaching ultrasound guided paracentesis is limited.

While the use of ultrasound in medicine has been shown to be useful, effective and teachable, formal training in ultrasound in internal medicine residency is not common as evidenced by the lack of available literature on ultrasound training. Internal medicine residents' experience and comfort level with ultrasound-guided invasive procedures also remains unclear. Our hypotheses are as follows: i. training residents in the use of ultrasound to perform clinically relevant procedures is feasible and will improve their ability to perform procedures during their training; ii. Improved ultrasound use will lead to less procedure related complications and enhanced patient safety. In this study, we sought to assess the feasibility of incorporating an ultrasound training workshop utilizing resident volunteers and cadaveric models during intern orientation and evaluate the comfort of first year internal medicine residents in using ultrasound to perform invasive procedures before and after the ultrasound training module.

## Methods

Our internal medicine residency program incorporates a mandatory procedure workshop during intern orientation. The workshop is led by subspecialty fellows, staff, and senior medical resident volunteers and utilizes cadaveric models to teach lumbar puncture, thoracentesis, and paracentesis [13]. This study incorporated an ultrasound training module into the procedure workshop during intern orientation in June 2009. The module consisted of a 40 minute didactic session, followed by 80 minutes of "hands-on" practice. [See additional file 1 for a diagrammatic representation of the study design.] The didactic session focused on principles of ultrasound imaging, machine controls and techniques of optimal image acquisition. It was taught by a board certified internal medicine physician who has also completed radiology residency. During the first 20 minutes of the hands-on session, residents were oriented to the ultrasound machine and its controls and practiced image acquisition. This was followed by the identification of anatomic structures including internal jugular vein and thyroid gland on resident volunteers. The next 60 minutes of the hands-on session took place in our institution's simulation center where residents practiced ultrasound guidance to perform thoracentesis and paracentesis on fresh non-embalmed cadavers with saline infused pleural and peritoneal spaces. The following machines were used during this workshop: SonoSite Titan with a C15 4-2 MHz transducer; SonoSite Micromaxx with C60e 5-2 MHz and L25e 13-6 MHz transducers; SonoSite S series and SonoSite M-Turbo with a C60x 5-2 MHz transducer. During the first 20 minutes of the hands-on session, residents practiced the use of ultrasound at two stations. The first station was dedicated to lung structures. The cadaver was in a seated position to mimic a real-life situation. Thoracentesis kits were provided and residents were asked to identify pleural space, lung tissue, and pleural effusion and attempt thoracentesis with ultrasound. The second station was dedicated to abdominal structures. The cadaver was in a supine position. Residents were asked to identify peritoneal space, ascetic fluid, bowel, and attempt paracentesis using ultrasound. The next 40 minutes were divided into two segments of 20 minutes. There were four stations; two stations dedicated to abdominal structures and paracentesis and two were dedicated to lung, pleural space, and thoracentesis. The abdominal stations were led by a gastrointestinal fellow and the lung stations were led by pulmonary and critical care fellows. The fellows were expected to teach the residents the indications, risks, and benefits of paracentesis and thoracentesis and educate them about the use of ultrasound to perform these procedures.

This study was approved by the Institutional Review Board. Residents completed anonymous pre and post-module surveys rating the following questions on a 5-point Likert scale (1 = extremely unconfident, 3 = neutral, 5 = extremely confident): 1) "How confident do you feel in your understanding of the principles behind the use of ultrasound technology to perform invasive procedures in internal medicine?" 2) "How confident are you that further training on ultrasound principles and techniques will increase your comfort level with ultrasound use for invasive procedures?" 3) "How comfortable do you feel identifying the following structures: lung, pleural effusion, bowel, peritoneal cavity, ascites, thyroid, and internal jugular vein on ultrasound?" Interns were also asked to rate their agreement with the following statements on a similar scale (1 = strongly disagree, 3 = neutral, 5 = strongly agree): 4) "Ready access to ultrasound equipment for residents on inpatient medical services would increase my comfort level with performing invasive procedures on the medical wards" and 5) "I anticipate performing invasive procedures such as thoracenteses and paracenteses frequently after residency graduation." Since surveys were anonymous, traditional paired t-tests were not possible. In order to be most conservative in our analysis, we systematically paired scores to minimize the mean difference and maximize the variability. This created the most unfavorable pairings with respect to our hypothesis, allowing us to calculate conservative upper bounds on the p-values for the unavailable paired t-tests. These conservative p-values were assessed for significance at the 0.01 level.

## Results

Seventy-five (99%) interns completed the pre-intervention survey. Mean (SD) confidence in understanding the use of ultrasound during invasive procedures was 2.63 (0.97) and in the ability of further training to increase comfort level was 4.55 (0.98) (Table 1). Mean (SD) comfort in identifying the seven structures ranged from 2.08 (1.06) for thyroid to 2.83 (1.10) for internal jugular vein. Mean (SD) agreement that access to ultrasound equipment on inpatient services would increase comfort level with invasive procedures was 4.45 (1.00) while anticipation of performing invasive procedures after residency it was 3.40 (1.30).

**Table 1:** Summary of Survey Responses

									Least Favorable		
		PRE			POST		Difference		Paired Difference		
		
Survey Question	n	Mean	SD	n	Mean	SD	Mean	SD	Mean	SD	P-value***
How confident* do you feel in your understanding of the principles behind the use of ultrasound technology to perform invasive procedures in internal medicine?	75	2.63	0.97	55	4.00	0.47	1.37	0.80	1.00	1.15	<.0001

How comfortable** do you feel identifying the following on ultrasound?											
**Lung**	75	2.52	1.04	61	4.00	0.61	1.48	0.88	1.13	1.34	<.0001
**pleural effusion**	75	2.61	1.03	61	4.16	0.55	1.55	0.85	1.25	1.31	<.0001
**Bowel**	75	2.45	1.08	61	3.64	0.78	1.19	0.96	0.85	1.62	0.0001
**peritoneal cavity**	75	2.51	1.13	61	3.61	0.84	1.10	1.01	0.75	1.74	0.0012
**Ascites**	74	2.81	1.13	61	3.82	0.79	1.01	0.99	0.67	1.62	0.0019
**Thyroid**	75	2.08	1.06	61	3.95	0.92	1.87	1.00	1.62	1.89	<.0001
**internal jugular vein**	75	2.83	1.10	61	4.48	0.62	1.65	0.91	1.34	1.53	<.0001

*Level of confidence rated on a 5 point Likert scale (1 = extremely unconfident, 3 = neutral, 5 = extremely confident)

**Level of comfort rated on a 5 point Likert scale (1 = extremely uncomfortable, 3 = neutral, 5 = extremely comfortable)

***conservative upper bound on traditional paired t-test

Following the intervention, 55 (72%) interns again rated their confidence in understanding the use of ultrasound, and the mean (SD) increased to 4.00 (0.47). Sixty-one (80%) interns again rated their comfort with identifying the seven structures, and mean (SD) comfort ranged from 3.61 (0.84) for peritoneal cavity to 4.48 (0.62) for internal jugular vein. Residents' confidence in identifying all seven anatomic structures showed an increase over the pre-intervention means (Table 1).

The conservative upper bounds for the paired t-test p-values showed a significant increase in confidence in the understanding of ultrasound principles (p < .0001) and significant increases in comfort identifying all seven structures (all p < .002).

## Discussion

To our knowledge, this study describes the first formal ultrasound training module as part of a required orientation for incoming internal medicine residents. Our results demonstrate that internal medicine residents recognize the need to perform ultrasound guided procedures in their future careers and desire further training in ultrasound techniques. Our ultrasound training module increased residents' self-reported comfort with the use of ultrasound for identification of selected anatomical structures and guided invasive procedures. We found the largest improvement in identifying the internal jugular vein, which has clinically relevant ramification when residents are attempting central line placement in critically ill patients, and in identifying the thyroid gland. This likely occurred because residents were asked to identify these structures on resident volunteers, thus allowing for a real-life image with the ultrasound. Residents comfort also increased for the identification of lung and pleural effusion, which may decrease the incidence of pneumothorax when bedside thoracentesis are done under ultrasound guidance. Interestingly, the smallest mean difference in resident confidence was in identification of peritoneal cavity and ultrasound guided paracentesis. We postulate that this occurred because of difficulty in replicating abdominal ascites in cadavers. The infused saline layered to the flanks and the loss of bowel gas made the separation of bowel and peritoneal fluid images more difficult to detect. These findings have important implications in patient care and highlight the need for creative ways to teach physicians how to acquire the skill of effective use of ultrasound imaging.

This study was conducted within a single, albeit large, internal medicine residency program therefore further research is needed to determine whether the results are generalizable to other training environments. Further, residents' confidence with ultrasound use was self-reported; we did not obtain an objective measure of residents' ultrasound skill. Finally, since data were anonymous traditional paired t-tests were not possible, we still identified significant and educationally meaningful differences between pre and post assessments using the most conservative and unfavorable pairings (i.e. worst case scenario) with respect to our hypothesis. Despite these limitations, this study highlights several important lessons. Implementation of an ultrasound training module is both feasible and initially effective. The effectiveness of an ultrasound module should be assessed by comfort level, knowledge, and skill over frequent time intervals throughout residency training. This study showed significant improvement in comfort level. Moreover, the use of cadavers was shown to be helpful in teaching ultrasound guided thoracentesis, but less effective in teaching paracentesis. Future study will incorporate objective knowledge and observed skill assessment at four month intervals, establish resident run ultrasound-guided paracentesis teams, and reassess the comfort of residents in performing ultrasound-guided procedures after an easily accessible ultrasound machine becomes available.

## Conclusions

An ultrasound training module can be incorporated as a mandatory component of physician orientation. Our study shows how an ultrasound training module can help prepare internal medicine residents to effectively use ultrasound for invasive procedures. Further study is needed to objectively assess knowledge retention and skill acquisition post-ultrasound training and investigate correlation between ultrasound trained physicians and improved procedural safety.

## Competing interests

All co-authors have reviewed this manuscript and agree with its content. This manuscript is not under review by any other publication. No financial or conflict of interest to disclose.

## Authors' contributions

AB was the main contributor to study conception and design. She developed and taught the didactic and hands-on session during orientation. She facilitated data collection and entry and provided background literature search, several substantial revisions to the manuscript, and approved the final version to be published. PYT assisted with statistical support, provided significant feedback on different sections of the manuscript, and approved final version to be published. AJH contributed to analysis and interpretation of data. He provided feedback on methods, and created table with results. DAR assisted with interpretation of results and provided feedback on multiple revisions and approved the final version to be published. MWC assisted in background literature search and design of the project. He helped with teaching and collection of the data. He contributed to the manuscript and approved the final version to be published. FHM assisted with statistical support and provided feedback on the manuscript. MTK assisted with background literature search, conception, and design of the project. She helped with teaching and collection of data. She created first draft and worked on multiple revisions and approved final version to be published. All authors read and approved this final manuscript.

## Pre-publication history

The pre-publication history for this paper can be accessed here:

http://www.biomedcentral.com/1472-6920/11/75/prepub

## Supplementary Material

Additional file 1 **Study Design Diagram**. A diagrammatic representation of the study design, detailing the workshop process. Click here for file
