# Supplementary material for: Effectiveness of an Ultrasound Training Module for Internal Medicine Residents
Source: BMC Med Educ. 2011 Sep 28;11:75. doi: 10.1186/1472-6920-11-75 (PMC3196730; doi:10.1186/1472-6920-11-75)
Supplement: Additional file 1 — Study Design Diagram. A diagrammatic representation of the study design, detailing the workshop process. [file 1472-6920-11-75-S1.PPT]

## Slide 1
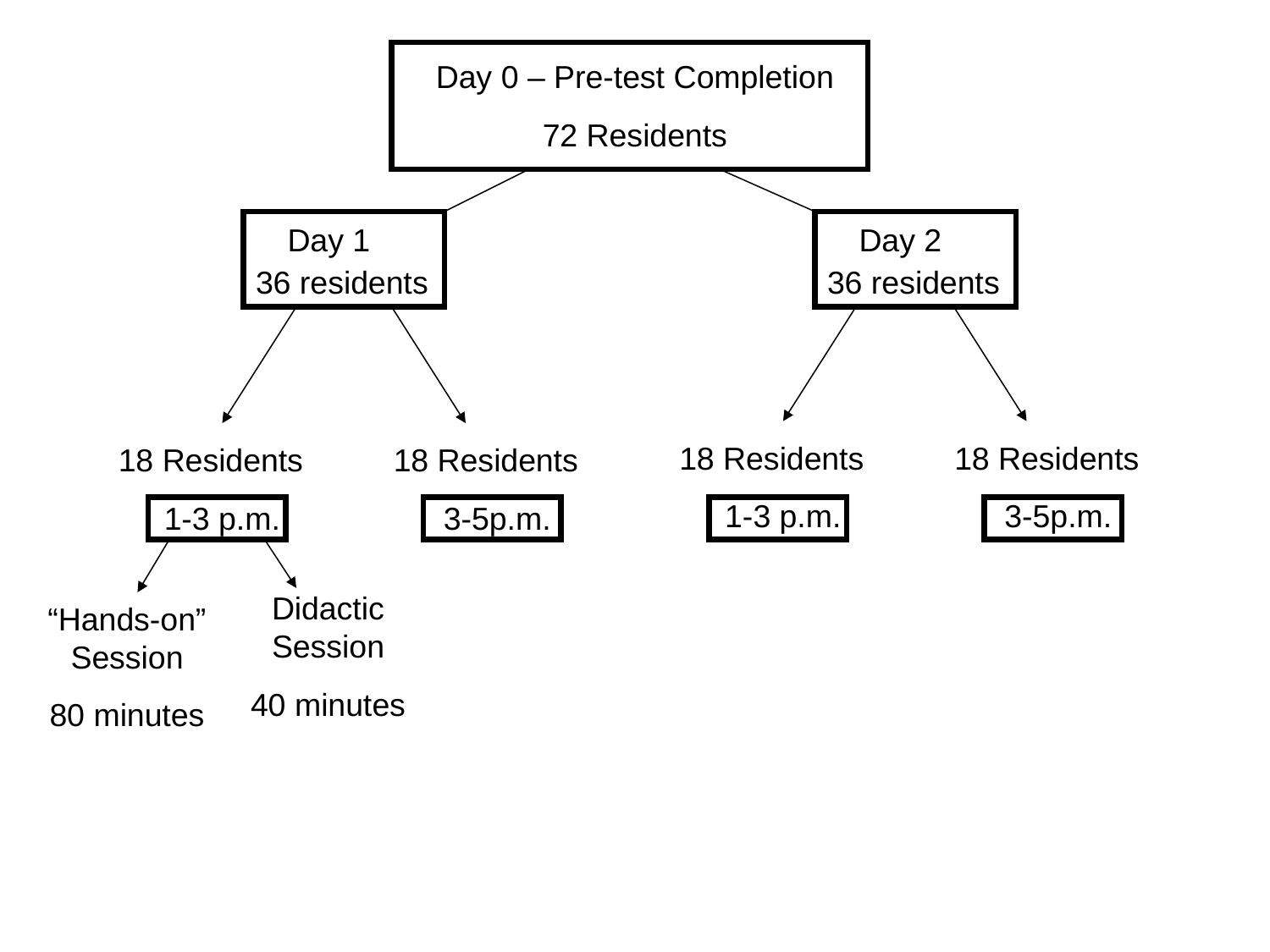

Day 0 – Pre-test Completion
72 Residents
Day 1
Day 2
36 residents
36 residents
18 Residents
1-3 p.m.
18 Residents
3-5p.m.
18 Residents
1-3 p.m.
18 Residents
3-5p.m.
Didactic Session
40 minutes
“Hands-on” Session
80 minutes

## Slide 2
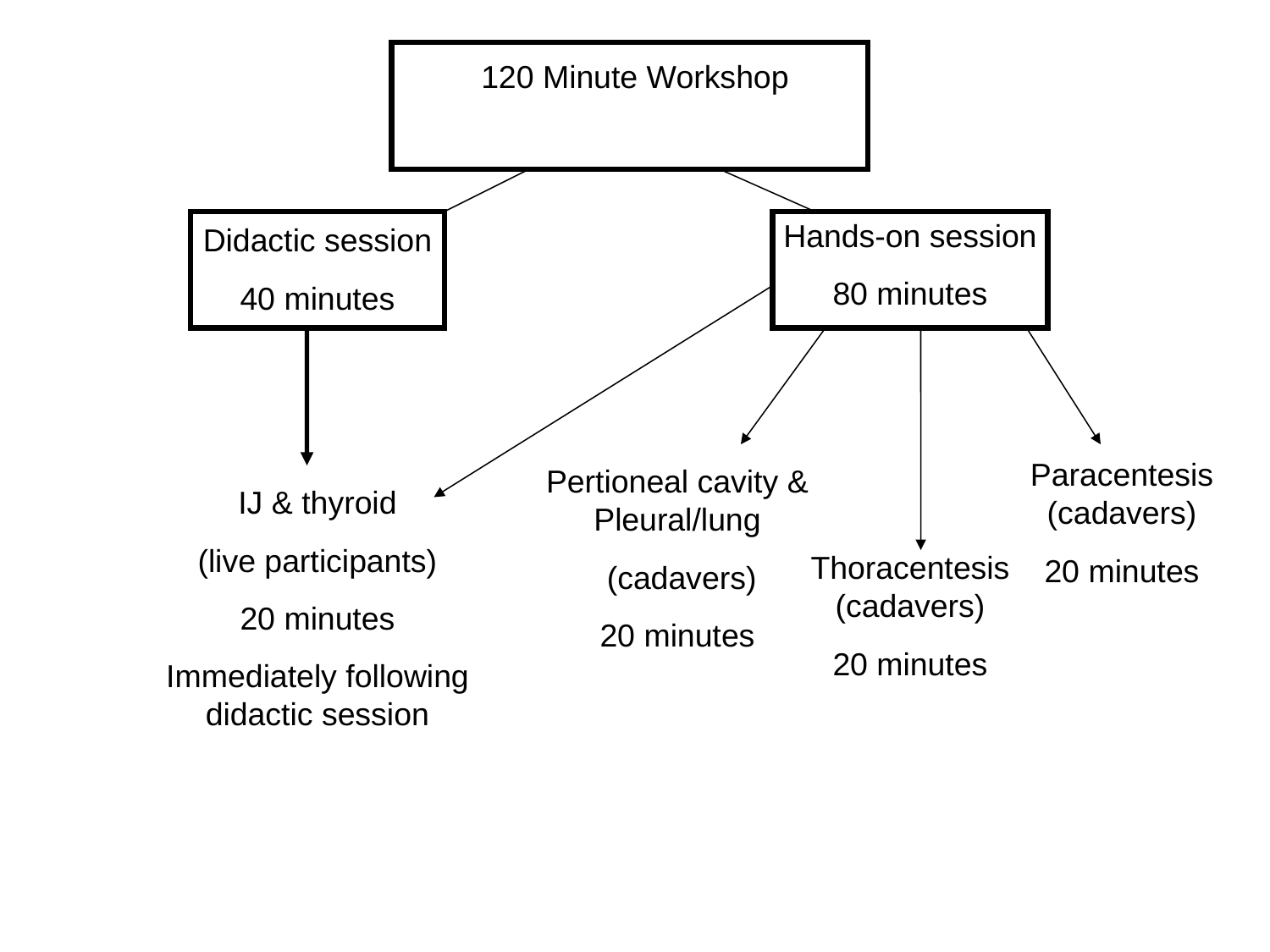

120 Minute Workshop
Hands-on session
80 minutes
Didactic session
40 minutes
Paracentesis (cadavers)
20 minutes
Pertioneal cavity & Pleural/lung
 (cadavers)
20 minutes
IJ & thyroid
(live participants)
20 minutes
Immediately following didactic session
Thoracentesis (cadavers)
20 minutes
